# CytroCell@Nafion: Enhanced Proton Exchange Membranes

**DOI:** 10.1002/gch2.202500338

**Published:** 2025-10-30

**Authors:** Daria Talarico, Enrica Fontananova, Teresa Sibillano, Rosaria Ciriminna, Stefania Palermo, Francesco Galiano, Gianluca Di Profio, Alberto Figoli, Giovanna Li Petri, Giuseppe Angellotti, Francesco Meneguzzo, Cinzia Giannini, Mario Pagliaro

**Affiliations:** ^1^ Istituto per la Tecnologia delle Membrane CNR Rende (CS) Italy; ^2^ Istituto di Cristallografia CNR Bari Italy; ^3^ Istituto per lo Studio dei Materiali Nanostrutturati CNR Palermo Italy; ^4^ Istituto per la Bioeconomia CNR Sesto Fiorentino (FI) Italy

**Keywords:** CytroCell, Nafion, nanocellulose, proton exchange membrane, water electrolysis

## Abstract

We investigate the use of lemon CytroCell nanocellulose as a new biobased filler for Nafion‐based proton exchange membranes. Pristine and composite membranes are prepared via casting and solvent evaporation technique. CytroCell is added up to 20 wt.% with respect to the polymer in a hydroalcoholic solution of Nafion perfluorosulfonic acid ionomer. Composite CytroCell@Nafion membranes are homogeneous on a molecular scale and showed enhanced proton conductivity with optimal performance for the composite membrane embedding 10 wt.% CytroCell. The composite membranes also showed improved flexibility and ductility compared to Nafion pristine membranes. Should stability of the new membranes be confirmed during prolonged PEM electrolyzer or H_2_ fuel cell operation, these findings open the route to the development of enhanced PEM membranes of broad applicability.

## Graphical abstract

A 10 wt.% amount of lemon CytroCell nanocellulose added to Nafion results in homogeneous composite CytroCell@Nafion membranes showing substantially higher proton conductivity, flexibility, and ductility than pristine Nafion membranes prepared via the same casting method and membrane post‐treatment. Should stability of the new membranes be confirmed during prolonged PEM electrolyzer or H2 fuel cell operation, these findings open the route to the development of enhanced PEM membranes of broad applicability.

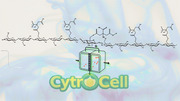

## 1 Introduction

Alkaline water electrolysis (AWE) mediated by low cost and durable nickel‐based electrodes in electrolysis cells operated at 80°C in 30% KOH is employed to produce about 1%–2% of the 55 million tonnes of H_2_ manufactured yearly across the world [1]. “Green” hydrogen obtained from water via electrolysis powered by electricity obtained from renewable (and intermittent) energy sources, particularly wind and sunlight, is a critically important energy storage technology [2]. Lowering the cost of “green” hydrogen requires to reduce the cost of the electrolytic process, which requires a substantial amount of electricity (55 kWh/kg H_2_), as well as lowering the cost of electrolyzers [3].

Though more capital‐intensive, polymer electrolyte membrane (PEM) water electrolysis, in which a cation exchange polymeric membrane acts both as gas separator and electrolyte for the selective proton transport, is the most efficient electrocatalytic route to hydrogen and oxygen [4].

Among the different PEMs, perfluorosulfonic acid (PFSA) membranes are currently those of highest performance in terms of hydrogen ion permeability and chemical stability [5].

State‐of‐the‐art PEM electrolyzers employ ionomeric PFSA membranes such as Nafion (a tradename of Dupont), Flemion (Asahi Glass), Aquivion (Solvay), and Aciplex (Asahi Kasei) [5].

Consisting of tetrafluoroethylene chains connected to perfluorovinyl ether groups terminated by sulfonate groups, Nafion is perhaps the best‐known fluorinated polymer used to make PFSA membranes. However, Nafion and the other PFSA ionomer membranes deteriorate over prolonged operation periods due both to mechanical and chemical degradation. Mechanical degradation is due to local changes in relative humidity and cyclic stress applied to the membrane, gradually reducing the membrane's mechanical strength, leading to defects such as poor interfacial contact between the membrane and the electrode and pinhole formation [6]. Chemical degradation is due to radical‐induced chemical decomposition in which ·OH or ·OOH radicals generated during PEM electrolyzer or fuel cell operation attack the backbone and side chains of the membrane or ionomer binder [7]. Moreover, PFAS membranes suffer of conductivity decay at temperature exceeding 80°C, particularly at low humidity levels, due to irreversible changes in the membrane's nanomorphology and in the states of water and ion–water channels that reduces proton transport [8]. In brief, besides enhanced modeling of degradation for prediction of electrolyzer and fuel cell durability [9], widespread uptake of PEM electrolyzers (and PEM fuel cells) for the production of affordable “green” hydrogen sourced from intermittent renewable energy sources requires substantial improvement of their current durability [10].

One of the most investigated strategies to improve the durability of PEMs is their combination with functional additives, affording composite membranes in which the polymer is combined with a relatively small amount of inorganic or organic additives [11].

Inorganic fillers employed due to formation of stable hydrophilic pathways for proton transport and enhanced water retention include silicates, metal oxides, nanotubes, and metal‐organic frameworks [12].

Moreover, amongst biobased additives, wood‐derived lignin and nanocellulose are particularly promising. Lignin‐based additives offer built‐in antioxidant functionality, scavenging peroxide radicals generated at the anode [13].

Nanocellulose has also been widely investigated as a filler able to enhance PFSA‐based membrane properties. For example, the addition of cellulose nanocrystals to Nafion at 5 wt.% loading, increases the water uptake and the thickness swelling, improves tensile strength, elongation at break, and Young's modulus [14]. The biodegradable and biobased nature of nanocellulose, furthermore, dramatically improves the environmental profile of the composite membrane. However, the relatively high cost of nanocellulose, largely due to extraction and fibrillation processes generating harmful effluents and requiring large energy amount, so far limited its widespread uptake in industrial applications [15].

This work advances nanocomposite Nafion membranes thanks to the use of a new nanocellulose imparted with unique chemical and physical properties, sourced at low cost from agro‐industrial waste. Obtained from industrial citrus processing waste (CPW) via hydrodynamic (HC) or acoustic (AC) cavitation carried out in water only and generating no harmful effluents, CytroCell consists of poorly crystalline submicron cellulose fibrils in which a substantial fraction of cellobiose units is esterified with citric acid [16]. Added in 1 wt.% amount to polymerizable ion liquid membranes, lemon CytroCell imparts the resulting composite membranes with pronounced mechanical strength and exceptional chemical stability in concentrated alkali, which is required for application of said composite membranes in AWE [17]. Expanding the applicability of this new nanocellulose to polymer electrolyte fuel cell and electrolyzer membranes, now we report that lemon CytroCell sourced both via HC or AC added in optimized 10 wt.% amount to Nafion affords highly stable CytroCell@Nafion membranes whose conductivity, flexibility, and ductility vastly exceed those of pristine Nafion membranes.

Another innovative aspect of the research is the use of a hydroalcoholic solution of Nafion, instead of toxic organic solvent such as dimethylformamide (DMF) and dimethylacetamide (DMA), to prepare membranes by casting method frequently used in state‐of‐the‐art membrane preparation protocols to obtain stable membranes. Although previously reported, the casting of Nafion membranes from a polymeric dispersion in mixture of alcohols and water at low temperature usually affords membranes that are less stable than those prepared using high boiling solvents like DMA or DMF [18]. This difference stems from the solvent ability to affect the polymer's morphology and macromolecular bonding during the casting process. Aprotic solvents promote better macromolecule bonding and optimal morphology, leading to stronger membranes with superior mechanical stability [18].

In this work, we demonstrate that by thermal and chemical post‐treatment of the CytroCell@Nafion membranes, it is possible to obtain stable and high‐performance Nafion composite membranes from substantially greener hydroalcoholic solutions.

## 2 Results and Discussion

### 2.1 Membrane Preparation

**FIGURE 1:**
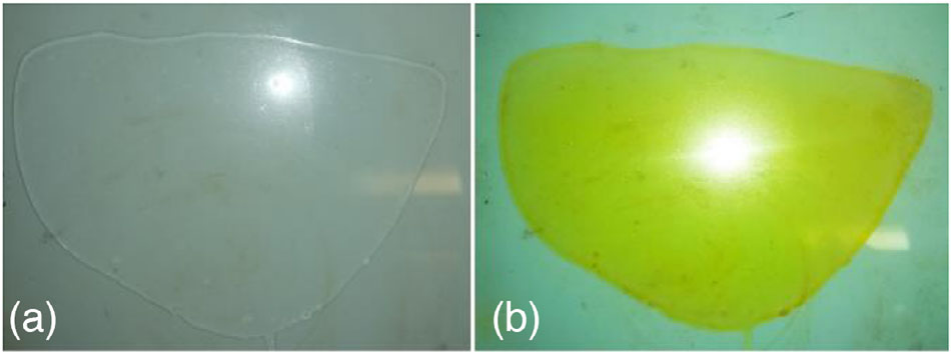
Unmodified Nafion (a) and composite CytroCell@Nafion (b) membranes containing 5 wt.% of CytroCell prepared via casting and solvent evaporation.

Pristine (Nafion pristine) and composite (CytroCell@Nafion) membranes were prepared via casting and solvent evaporation using a hydroalcoholic solution without any toxic organic solvent via the nonsolvent‐induced phase separation method, namely the most widely process employed to fabricate asymmetric structures in industrial standard flat‐sheet and hollow fiber membranes [19]. Photographs in Figure 1 show evidence that both pristine Nafion and composite CytroCell@Nafion membranes obtained were homogeneous, transparent, flexible without any visible aggregates and precipitates.

The bright yellow color of the CytroCell@Nafion membrane is due to citrus flavonoids abundant in lemon CPW that are effectively extracted during the cavitation‐based extraction process, during which also highly bioactive citrus IntegroPectin is obtained in the aqueous phase [20].

**FIGURE 2:**
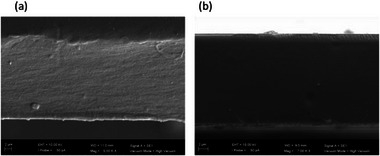
SEM images of (a) Nafion_pristine and (b) CytroCell@Nafion (10 wt% in CytroCell) membranes cross‐section.

The homogeneous appearance of both hybrid membranes points in each case to excellent dispersion of CytroCell in the polymeric matrix. Confirming excellent dispersion also on the microscale, the scanning electron microscopy (SEM) photographs of the unmodified Nafion and composite membranes (Figure 2) clearly show a dense structure for both unmodified Nafion and composite membranes.

**FIGURE 3:**
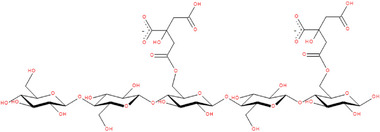
Chemical structure of CytroCell.

A cellulose citrate ester with about 40% degree of esterification, lemon CytroCell exhibits amphiphilic nature due to the presence of chemically bound citrate groups, along with hydrophilic ─OH and ─COOH groups. Figure 3 shows the lemon CytroCell structure corresponding to the first β‐1‐4 linked D‐glucopyranose (β‐Glcp) residues, two of which were esterified to achieve a substitution degree of 40%, measured by DRIFT spectroscopy [21].

Further molecular dynamics simulation studies including solubility, association types, and clustering phenomena calculations employing the structure in Figure 3, unveiled both excellent solubility in polar solvents including water along with strong chemical stabilization promoted by interaction of charged citrate groups [21].

### 2.2 Conductivity, Permselectivity, and Mechanical Properties

**FIGURE 4:**
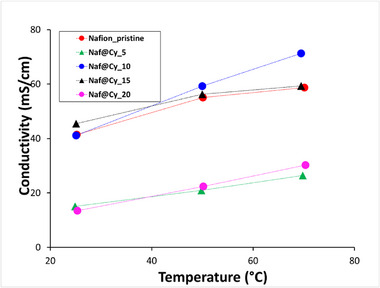
In‐plane conductivity of membranes: pristine (Nafion_pristine) and composite CytroCell@Nafion membranes containing CytroCell at 5 wt.% (Naf@Cy_5), 10 wt.% (Naf@Cy_10), 15 wt.% (Naf@Cy_15), and 20 wt.% (Naf@Cy_20) load.

Characterized via electrochemical impedance spectroscopy (EIS) to determine in‐plane conductivity, all CytroCell@Nafion membranes showed a significant increase for proton conductivity with rising temperature (Figure 4).

Further inspection of data obtained from proton conductivity measurements of different membranes containing different loads of incorporated bioadditive (5, 10, 15, and 20 wt.%) shows that the incorporation of 10 wt.% CytroCell maximizes proton conductivity, reaching values higher than those of pure Nafion membrane already at 50°C, and further growing with increasing temperature.

In composite membranes, the hydroxyl, carboxyl, and ester hydrophilic groups of CytroCell establish a network of hydrogen bonds with the PFSA structure. Similar to the structure of PFSA membranes, in a hydrated environment the hydrophilic groups interact to form channels that facilitate H_2_O molecule and proton transport by Grotthus or vehicular mechanism [22].

Optimal interaction between Nafion functional groups and the cellulosic material depends on several factors, such as, van der Waals forces, steric repulsion, and electrostatic interactions [23]. The carboxylated nanofibers of CytroCell can promote long‐range inter‐connections between the polymeric networks, ensuring good performance even at elevated temperatures. Likely, formation of hydrogen bonds between the hydroxyl and carboxylic groups of the CytroCell nanocellulose and the sulfonic acid groups of the polymer ensures continuous channels for proton transfer. However, a low loading of the additive (5 wt.%) or an excessive one (20 wt.%) had a negative effect on the membrane performance for complementary reasons.

An overly low CytroCell content (5 wt.%) is not sufficient to reach the percolation threshold required to ensure formation of a good interconnection between the two components of the membrane (the polymer and the CytroCell nanofibers). On the contrary, an excessive load of CytroCell (20 wt.%) likely leads to the partial aggregation of filler nanoparticles, hindering proton mobility.

**FIGURE 5:**
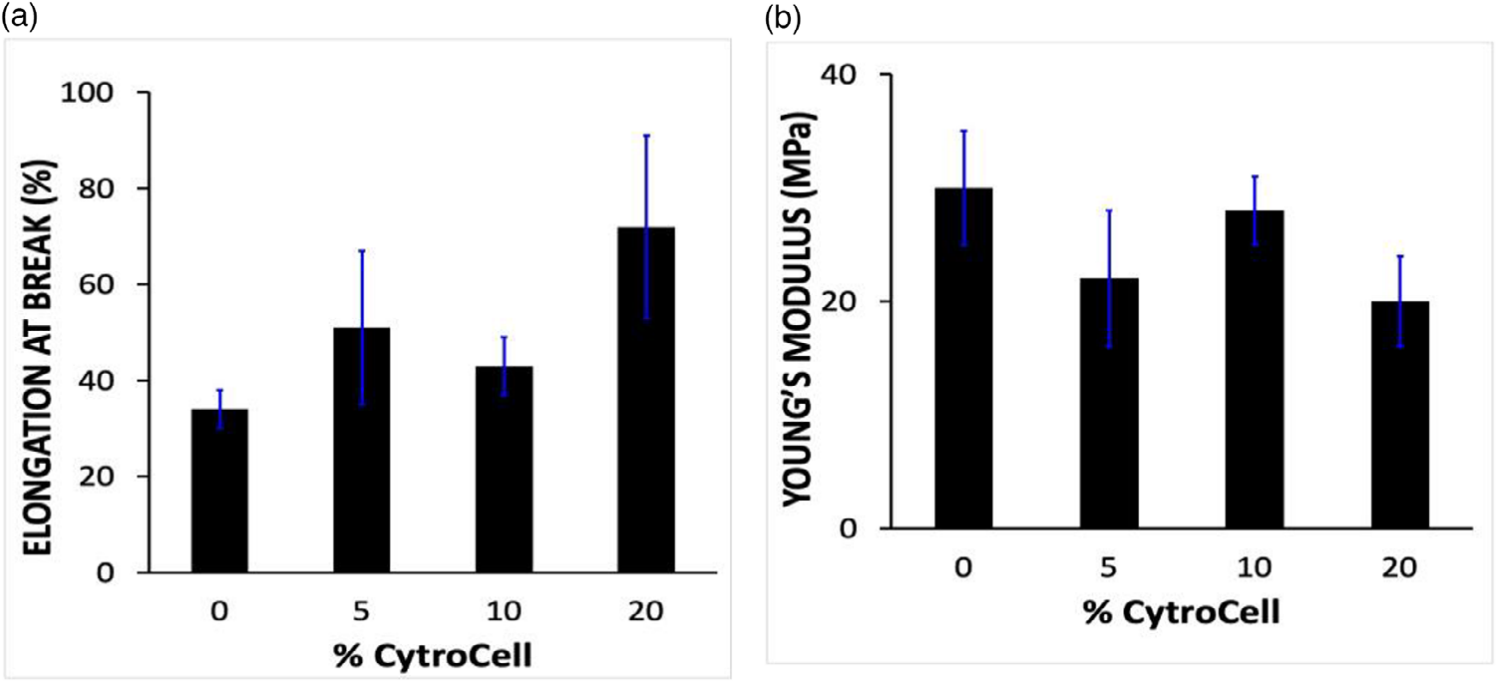
Elongation at break (a) and Young's modulus (b) for Nafion and CytroCell@Nafion composite membranes vs. CytroCell wt% load in membrane.

On the other hand, increasing the CytroCell load in the CytroCelll@Nafion membranes resulted in increased elongation at break (Figure 5a). The effect is due to well‐known reinforcing effect of nanocellulose fibers in nanocellulose‐polymer composites, enhancing the ability of the composite membrane to stretch before breaking [24]. Furthermore, the formation of a more entangled or interconnected microstructure, facilitated by the amorphous nature of the CytroCell bionanomaterial, may contribute to greater energy dissipation during deformation, resulting in increased elongation before failure [25].

Conversely, the addition of CytroCell to the pristine Nafion membranes tends to reduce the membrane stiffness as evidenced by the Young's modulus trend with increasing CytroCell loads (Figure 5b).

Being a highly hydrated and dispersible form of nanocellulose [21], CytroCell nanocellulose may promote better stress distribution throughout the membrane improving interfacial interactions with the PFSA polymer chains. This can reduce stress concentrations and delay the onset of fracture, leading to improved mechanical ductility.

The presence of CytroCell, while enhancing proton conductivity and mechanical properties, might also introduce additional pathways for ion migration, thereby compromising the membrane's selective ion transport characteristics with consequent reduction of membrane permselectivity (Table 1).

**TABLE 1:** Permselectivity of pristine Nafion and composite CytroCell@Nafion membranes in 0.1/0.5 M NaCl at 25°C ± 1°C.

CytroCell (wt.%)	Permselectivity (%)
0	63.7 ± 0.5
5	51.5 ± 0.1
10	36.0 ± 0.3
15	56.1 ± 0.6
20	29.9 ± 0.9

The role and effects of thermal treatments—often employed as part of membrane conditioning protocols—were also investigated. From a structural and chemical perspective, thermal membrane treatment under dry conditions can lead to the so‐called “shrunk” state, characterized by reduced water uptake, increased polymer chain packing, and consequent decrease in proton conductivity. However, thermal treatment remains essential to enhance the mechanical stability of PFSA membranes and to maintain their dimensional integrity during handling and testing [26].

Hence, we examined the influence of the thermal treatment and successive chemical activation in acid solution. In‐plane proton conductivity measurements revealed that thermal treatment was essential to obtain mechanically stable membranes, with membranes without thermal treatment being fragile and easily broken during handling or long‐term storage in water. On the contrary, the Nafion‐based membranes after thermal treatment were mechanically stable.

**FIGURE 6:**
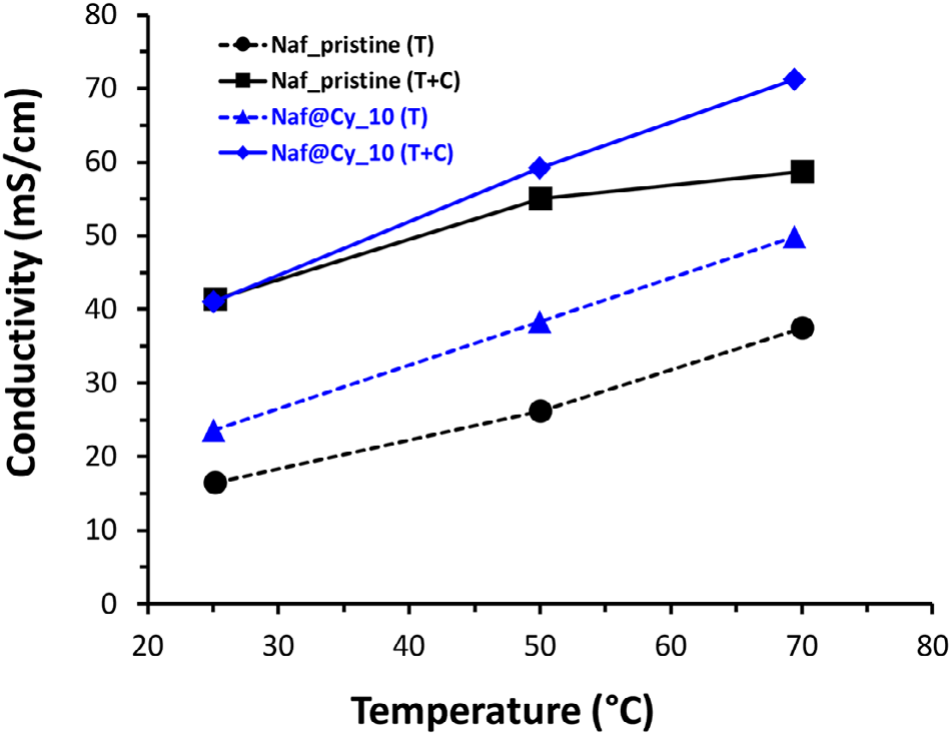
In‐plane conductivity of membranes: Nafion_pristine and CytroCell@Nafion (10 wt.% CytroCell load after thermal (T) and thermal plus chemical (T + C) treatments.

Addition of CytroCell to the Nafion membranes contributes to the preservation of ion‐exchange group activity within the composite membrane, as shown by the higher conductivity values compared to the pristine membrane after thermal treatment (Figure 6).

However, acid treatment of the membranes was necessary to further activate the functional groups by hydration and protonation. The conductivity was higher for the membrane containing 10 wt.% biobased filler for both treatments, thermal and thermal plus chemical, suggesting a synergistic interaction between CytroCell and the PFSA polymer matrix that enhances proton transport efficiency.

In brief, the highly flexible citrate groups present in the cellobiose units on lemon CytroCell [18], exert multiple positive effects justifying these findings: (i) they stabilize the cellulose polymer via specific interactions between the citrate groups and certain hydroxyl groups of the glycosidic backbone; (ii) ensure repulsion of the negatively charged nanofibrils that readily disperse in polar solvent; and, (iii) enhance conductivity thanks to their large negative charge originating from the carboxylate groups.

### 2.3 WAXS Investigation

**FIGURE 7:**
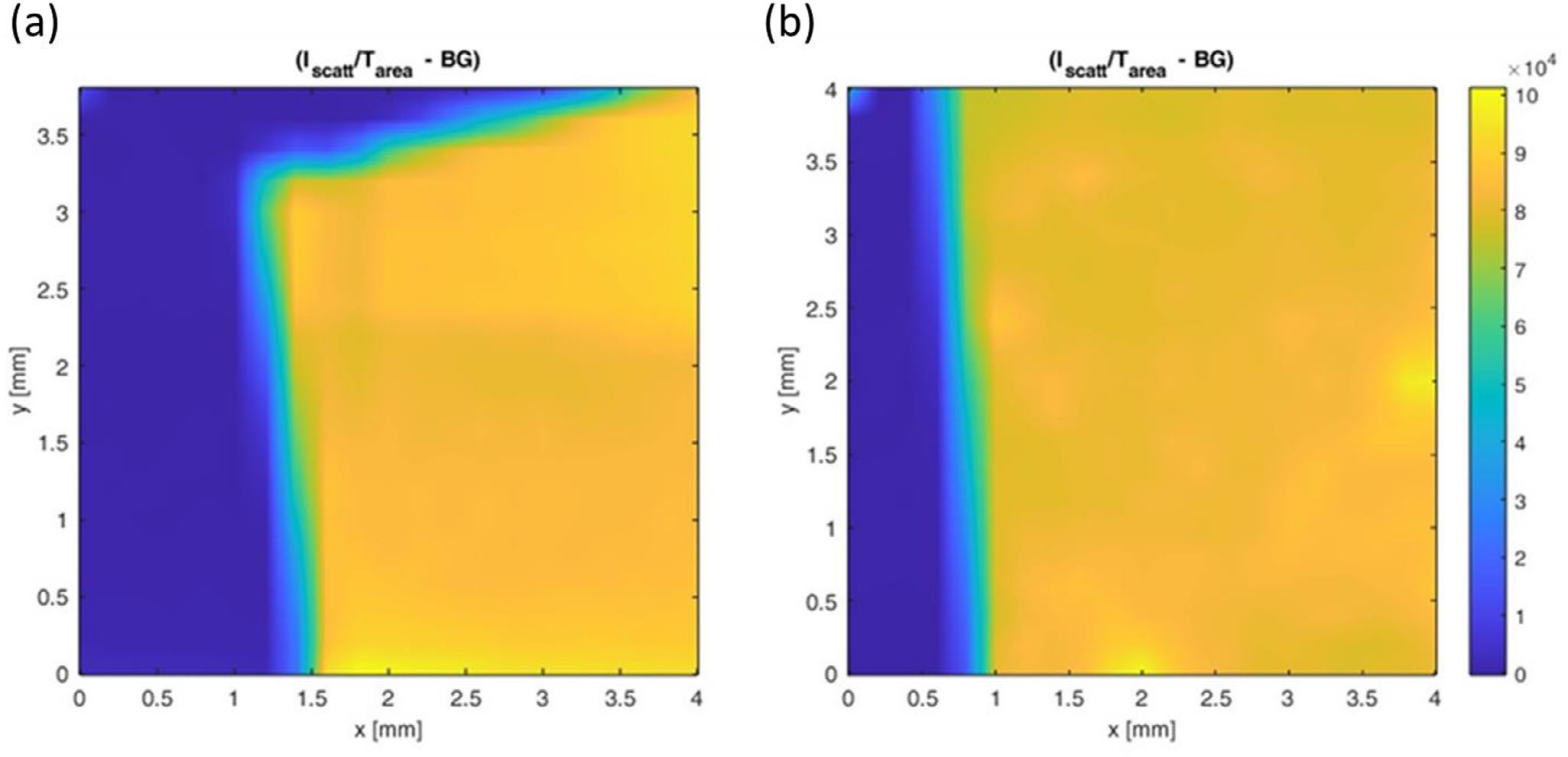
WAXS microscopy maps of (a) Nafion and (b) CytroCell@Nafion membranes.

As detailed in the Experimental section, preliminary WAXS (wide angle X‐ray scattering) measurements were performed using a sample‐to‐detector distance (SDD) of 41 cm and a Triton photon‐counting digital detector. This configuration enables spatially resolved reconstruction of inhomogeneities in the average electron density caused by the presence of CytroCell embedded in the membrane, across the entire illuminated area of the sample. The primary objective of the WAXS microscopy analysis was to assess the uniformity of CytroCell distribution within the Nafion membrane. WAXS microscopy maps (Figure 7) showed homogeneous scattering for both pristine Nafion membranes and those containing CytroCell.

Azimuthal integration of the scattered intensity over the entire analyzed area revealed an uniform scattering intensity and the absence of significant short‐range domains within the probed q‐range. Moreover, an increase in the amorphous scattering intensity was observed under identical measurement conditions, suggesting a higher degree of disordered structure of the composite membranes embedding the CytroCell nanofibers.

To further examine the presence of crystalline domains of CytroCell embedded within the membranes, WAXS measurements were performed by reducing the sample‐to‐detector distance to 10 cm. This setup allowed access to higher scattering angles, thereby enabling the detection of features associated with short‐range structural order. For each sample, WAXS data were obtained by azimuthally integrating the scattering signal over the full illuminated area, thus yielding the spatially averaged WAXS profile. The diffraction profiles were compared with those of a CytroCell powder reference sample, measured under identical experimental conditions, to allow the identification of the characteristic reflections of crystalline cellulose. For the WAXS measurements of CytroCell powder, the sample was sealed in an Ultralene sachet to prevent sample loss and then mounted in the beam path for direct exposure. Measurements were therefore carried out under the same experimental setup used for the membrane samples.

**FIGURE 8:**
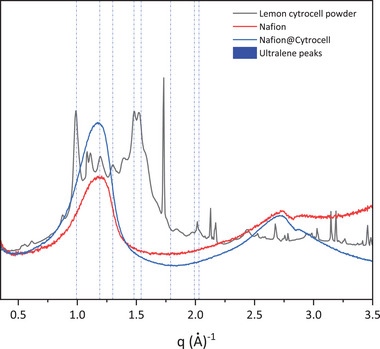
WAXS profiles of CytroCell powder (black line), Nafion@Cy_5n membrane (blue line), and pristine Nafion membrane (red line). Blue vertical lines indicate the positions of the diffraction peaks associated with the Ultralene sachet.

As shown in Figure 8, which compares the 1D WAXS patterns collected from the two membranes and the CytroCell powder, no distinct diffraction peaks are observed in the membrane containing CytroCell. This finding corroborates the WAXS microscopy observations and indicates that, upon dispersion in hydroalcoholic solution together with Nafion, the lemon‐derived CytroCell undergoes complete amorphization.

CytroCell nanofibers, originally organized as loosely aggregated, poorly crystalline cellulose microfibrils with lengths of 0.5–3 µm and widths of 110–420 nm [27], become well‐dispersed due to the strong electrostatic repulsion imparted by the negatively charged citrate groups on their surface.

This electrostatic stabilization prevents re‐aggregation and promotes uniform dispersion within the polymer matrix. Such behavior is critical for the formation of mechanically robust nanocomposites, as it allows the cellulose nanofibers to reach the percolation threshold at low loading levels [24], where a continuous, rigid nanofibrillar network forms throughout the composite material [28].

The WAXS pattern of lemon‐derived CytroCell exhibits multiple diffraction rings, including both sharp and broad peaks, indicative of the presence of multiple crystalline phases in addition to cellulose. Qualitative and semi‐quantitative analysis (see Figure S1) reveals that the pattern contains contributions from various cellulose polymorphs—predominantly cellulose Ia, Ib, and cellulose II—commonly found in cellulose extracted from citrus fruit peels.

Poor in lignin and rich in cellulose and hemicellulose, the peel of *Citrus* fruits, indeed, is an excellent source of microcrystalline cellulose or nanocellulose [29]. We ascribe the presence of the narrower peaks in the WAXS profile to larger crystals of hemicellulose, whose presence has been lately unveiled in lemon CytroCell by a detailed DRIFT analysis [21].

**FIGURE 9:**
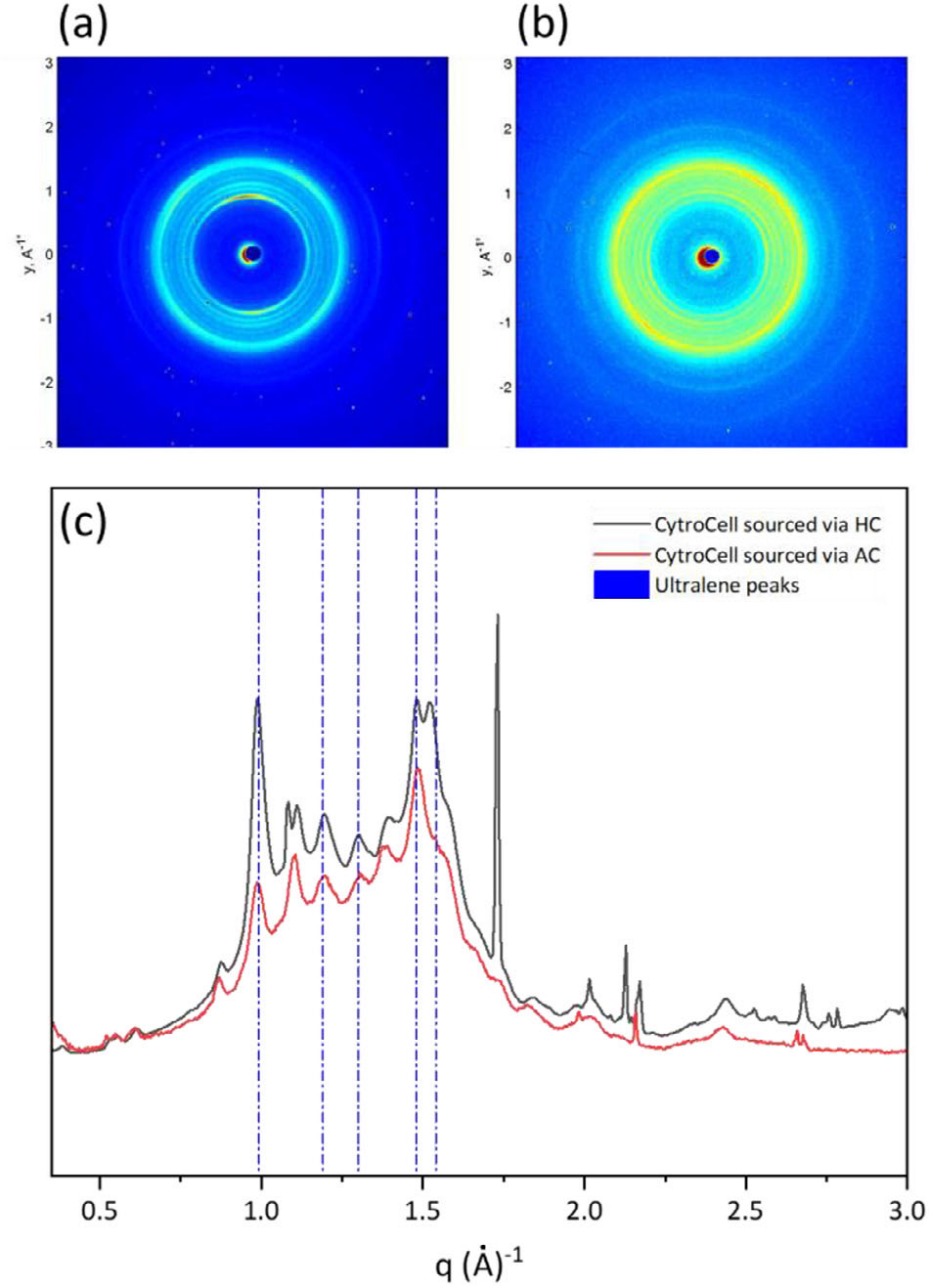
2D WAXS patterns of CytroCell obtained via hydrodynamic cavitation (HC) (a) and acoustic cavitation (AC) (b). (c) 1D WAXS profiles of both biomaterials.

The WAXS profiles of CytroCell obtained via acoustic cavitation (AC, red curve in Figure 9c) and hydrodynamic cavitation (HC, black curve in Figure 9a) exhibit similar crystalline structures, with nearly complete overlap of the diffraction signals (Figure 9), indicating comparable bulk crystallinity. In the HC‐derived sample, the appearance of a few additional discrete diffraction peaks suggests formation of secondary crystalline phases.

**FIGURE 10:**
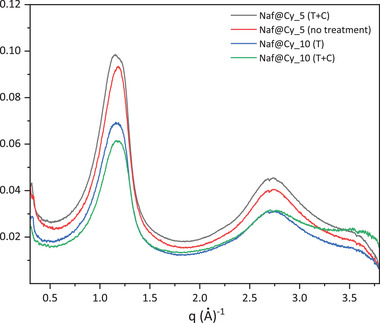
WAXS patterns of CytroCell@Nafion membranes containing 5 wt.% CytroCell without any treatment (red curve); 10 wt.% CytroCell, after thermal treatment (T, blue curve); 10 wt.% CytroCell, after combined thermal and chemical treatment (T + C, black and green curves).

However, the WAXS analysis of the membranes prepared with AC‐derived CytroCell under various processing conditions and concentrations also reveals in this case the disappearance of cellulose crystalline peaks (Figure 10).

This loss of crystallinity may be attributed to the dissolution of the bionanomaterial in the hydroalcoholic polymeric solution, rather than to thermal or chemical modification of the membranes, as confirmed by the similar WAXS profiles recorded for treated and untreated CytroCell@Nafion composite membranes.

These findings are directly relevant to forthcoming applications because PFSA‐based membranes for PEM electrolyzers and fuel cells require a high degree of stretchability and the capability to deform under stress without breaking, while the membrane is subject to anisotropic swelling during operations at high temperatures and various humidification levels [9]. State‐of‐the‐art PFSA membranes in commercial PEM fuel cells indeed are reinforced with a microporous expanded polytetrafluorethylene (ePTFE) thin film to obtain a ionomer composite membrane possessing greater dimensional stability and improved water distribution [30].

## 3 Conclusions

In conclusion, we have discovered that CytroCell nanocellulose sourced via cavitation (acoustic or hydrodynamic) of industrial lemon processing waste conducted in water only can be successfully employed to form CytroCell@Nafion membranes characterized by enhanced proton conductivity, and higher flexibility and ductility than pristine Nafion membranes.

In addition to improving the mechanical properties, the optimized 10 wt.% CytroCell loading significantly enhances also the proton conductivity of the composite CytroCell@Nafion membrane with respect to the pure polymeric membrane at high temperatures, starting from 50°C.

Here demonstrated for Nafion, these findings are general and can be extended to other PFSA polymers used to prepare membranes for PEM fuel cells and electrolyzers, because amphiphilic lemon CytroCell can be readily dispersed in water and in aqueous polar solvent mixtures.

Should stability of the new CytroCell@Nafion membranes be confirmed during prolonged PEM electrolyzer or H_2_ fuel cell operation, these outcomes open the route to the development of enhanced proton exchange membranes for electrolyzers and fuel cells based on PFSA polymers functionalized with new nanocellulose sustainably sourced from citrus processing waste, available at little cost and large amount in all countries where *Citrus* fruits are harvested.

No acid, base, or organic solvent is employed, and no energy‐intensive mechanical treatment of cellulose is required to fibrillate the cellulose (or partly carboxylated cellulose) fibers as in conventional nanocellulose production routes. Finally, a green solvent mixture (propanol, ethanol, and water) was used to prepare the composite membranes via the phase‐inverse technique. The solvent can be readily recovered and reused in subsequent membrane preparations thereby establishing a circular production process that extends from that of the CytroCell to its composite membranes for energy applications.

## 4 Experimental Section

### 4.1 CytroCell Preparation

Lemon CytroCell was prepared via HC or AC‐based extraction processes as previously described [16, 31], from industrial lemon processing waste kindly donated by a citrus processing company based in Sicily (OPAC Campisi, Siracusa, Italy).

### 4.2 Membrane Preparation and Characterization

Nafion membranes were prepared from a commercial 20 wt.% Nafion PFSA dispersions in propanol, ethanol, and water (D2020, QuinTech, Germany). The dispersion was cast on glass plate at 350 µm thickness and evaporated at 60°C. If not otherwise specified, the membrane was thermally treated at 150°C, activated in H_2_SO_4_ 1 M and finally rinsed in water until neutrality. Membranes obtained were stored in water at room temperature until use. Composite membranes were prepared following a similar protocol, but adding to the Nafion dispersion the CytroCell nanocellulose at loads varying from 5 to 20 wt.% with respect to the polymer dispersed by magnetic stirring.

Scanning electron microscopy (SEM) was carried out with an EVO MA10 microscope (Carl Zeiss, Oberkochen, Germany). Membrane cross sections were fractured in liquid N_2_ and coated with a thin gold layer before the analysis using a Sputtering Quorum Q 150R S device.

Electrochemical impedance spectroscopy (EIS) experiments were carried out with an Autolab PGSTAT302N potentiostat/galvanostat equipped with a frequency response analyzer module in the frequency range from 100 000 to 1 Hz with a 10 mV signal amplitude (Metrohm Autolab, Utrecht, the Netherlands). A four‐electrode configuration was used to measure the in‐plane conductivity in liquid water at 25°C, 50°C, and 70°C using a BT‐112 BekkTech conductivity cell (Scribner, Southern Pines, NC, USA). EIS spectra were processed with the Metrohm Autolab Nova 1.9.16 software.

The membrane permselectivity was calculated as the ratio of the measured potential across the membrane separating two solutions of different concentration (NaCl 0.1 and 0.5 mol/L at 25°C ± 3°C) and the theoretical value (37.8 mV). The membrane potential was measured by a digital multimeter, Fluke 289/FVR/IR3000 (Fluke Europe, Eindhoven).

The mechanical properties of the membranes in wet state were assessed in stress‐strain elongation experiments using a ZwickiLine Z2.5 testing machine (Zwick/Roell, Genova, Italy).

The WAXS characterizations were performed using a Rigaku (Akishima‐shi, Tokyo, Japan) system equipped with a three‐pinhole camera (300, 150, and 500 µm apertures in a high‐flux configuration), Fr‐E+ SuperBright rotating copper anode microsource (45 kV/55 mA; Cu Kα, λ = 0.15405 nm, 2475 W), and Confocal MaxFlux (CMF15‐105) focusing optics.

Free‐standing membranes of Nafion and CytroCell@Nafion were used for the investigation. These membranes were mounted in the sample holder with their surfaces perpendicular to the incident X‐ray beam. Scanning WAXS and X‐ray transmission microscopies covered a sample area of 4 mm × 4 mm, with measurements taken at 0.2 mm intervals. The beam footprint was approximately 0.2 mm in diameter. The transmitted X‐ray signal at each scan position was recorded by a photodiode integrated into the beamstop. The experimental setup featured a sample‐to‐detector distance of 41 cm for WAXS microscopy, limiting the effective *q*‐range to 0.1–1 Å^−1^ in reciprocal space.

WAXS data collected at each position were assembled into 2D maps using the SUNBIMs Supramolecular and Submolecular Nano‐ and Biomaterials X‐ray Imaging) custom software [32]. The processing steps included angular calibration, dark current subtraction, and absorption correction by normalizing WAXS intensities to the transmission coefficient (T). This yielded a spatial representation of the scatterer distribution integrated over the sample thickness.

To access the higher WAXS regime, the sample‐to‐detector distance was reduced to approximately 10 cm, providing a q‐range of about 0.3–3.5 Å^−1^. This corresponds to a real‐space resolution from ∼1.8 to ∼21 Å, allowing the detection of short‐range order and potential crystalline features. WAXS data were acquired by raster scanning the entire sample area, and WAXS signals from all positions were summed to obtain an integrated diffraction pattern.

Detector‐to‐sample distances were calibrated using a Si NIST standard powder for WAXS and an Ag‐behenate standard for SAXS measurements, respectively. The system is equipped with two distinct detectors: a Triton 20 gas filled proportional counter (1024 × 1024 array, 195 mm pixel size) for WAXS acquisition at 41 cm, and an image‐plate (IP) detector 250 × 160 mm in size, with 50‐ or 100‐mm effective pixel size (depending on binning), and off‐line RAXIA reader to collect WAXS data at 10 cm.

## Funding

The work of D.T., E.F., T.S., R.C., F.G., G.D.P., A.F., C.G., R.C., and M.P. was partially funded by the Ministero delle Imprese e del Made in Italy under the Piano Operativo della Ricerca “Ricerca e sviluppo sull'idrogeno” financially supported by the European Union ‐ NextGenerationEU ‐ M2C2 Investment 3.5, in the framework of the project PNRR Ricerca e Sviluppo sull'Idrogeno 2022‐2025 ‐ Accordo di Programma “Idrogeno” (PRR.AP015.017.002), “Obiettivo 1 ‐ Produzione di idrogeno verde e pulito”, LA 1.1.6 and 1.1.7. The work of E.F. and S.P. was partially supported by Ministero dell'Ambiente e della Sicurezza Energetica in the framework of the Project PERMANENT (Advanced materials and components for PEM fuel cells with innovative multi‐scale structuring for enhanced durability and stability), Progetti di Ricerca per l'Idrogeno PNRR – M2C2 investment 3.5 (RSH2A000012). The work of G.L.P. and T.S. was supported by MICS (Made in Italy ‐ Circular and Sustainable) Extended Partnership and received funding from the European Union Next‐GenerationEU (PNRR ‐ Mission 4 Component 2, Investment 1.3 ‐ D.D.1551.11‐10‐2022, PE00000004). The work of G.A. was supported by the SAMOTHRACE (Sicilian Micro and Nano Technology Research and Innovation Center) Innovation Ecosystem using funding from the European Union NextGeneration EU (PNRR – Mission 4 Component 2, Investment 1.5 (ECS00000022)). M.P. and R.C. thank Ministero dell'Università e della Ricerca for funding under project “FutuRaw ‐ Le materie prime del futuro da fonti non‐critiche, residuali e rinnovabili”, Fondo Ordinario Enti di Ricerca, 2022, CNR (CUP B53C23008390005).

## Conflicts of Interest

The authors declare no conflicts of interest.

## Supporting information




**Supporting file**: gch270062‐sup‐0001‐SuppMat.docx

## Data Availability

The data that support the findings of this study are available from the corresponding author upon reasonable request.
