# Supplementary material for: CytroCell@Nafion: Enhanced Proton Exchange Membranes
Source: Glob Chall. 2025 Oct 30;9(12):e00338. doi: 10.1002/gch2.202500338 (PMC12697083; doi:10.1002/gch2.202500338)
Supplement: Supplementary file 1 — Supporting file: gch270062‐sup‐0001‐SuppMat.docx [file GCH2-9-e00338-s001.docx]

CytroCell@Nafion: Enhanced Proton Exchange Membranes

**Daria Talarico**,**^1*^**[
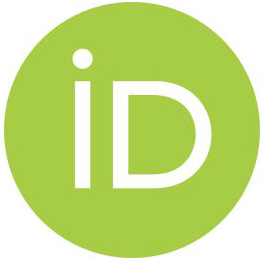
](https://orcid.org/0009-0007-2374-1484) **Enrica Fontananova**,**^1*^**[
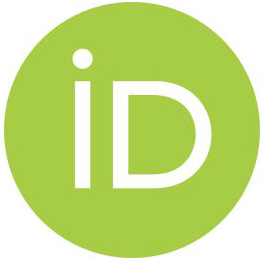
](https://orcid.org/0000-0002-2506-0160) **Teresa Sibillano**,**^2*^**[
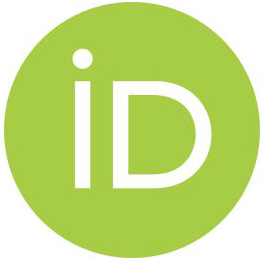
](https://orcid.org/0000-0002-7741-1436) **Rosaria Ciriminna,^3*^**[
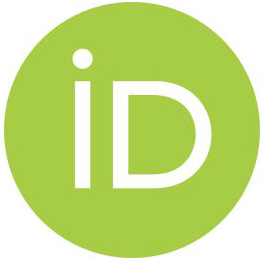
](https://orcid.org/0000-0001-6596-1572) **Stefania Palermo**,**^1^**[
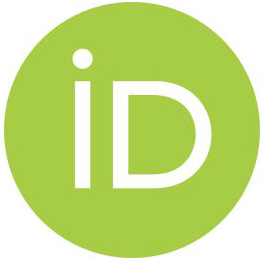
](https://orcid.org/0009-0000-6464-4364) **Francesco Galiano**,**^1^**[
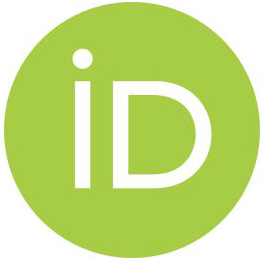
](https://orcid.org/0000-0003-0536-552X) **Gianluca Di Profio**,**^1^**[
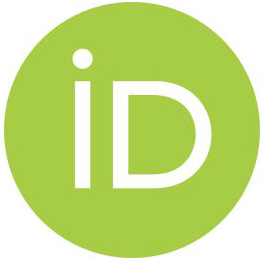
](https://orcid.org/0000-0002-8317-6233) **Alberto Figoli**,**^1^**[
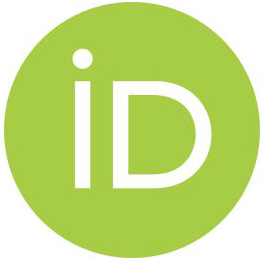
](https://orcid.org/0000-0002-3347-0506) **Giovanna Li Petri**,**^3^**[
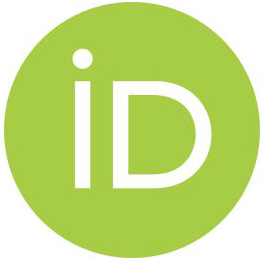
](https://orcid.org/0000-0001-6113-5978) **Giuseppe Angellotti**,**^3^**[
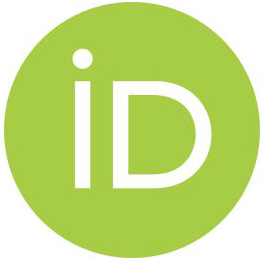
](https://orcid.org/0000-0001-9226-4748) **Francesco Meneguzzo**,**^4^**[
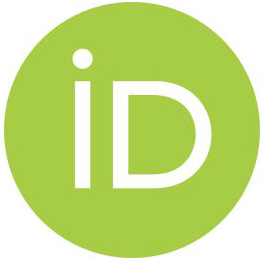
](https://orcid.org/0000-0002-5952-9166) **Cinzia Giannini**,**^2*^**[
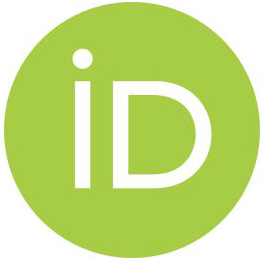
](https://orcid.org/0000-0003-0983-2885) and **Mario Pagliaro^3*^**[
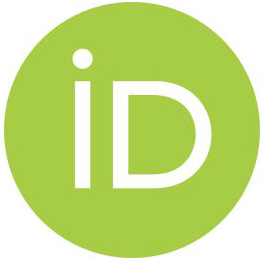
](https://orcid.org/0000-0002-5096-329X)

*^1^Istituto per la Tecnologia delle Membrane, CNR, via P. Bucci, cubo 17/c, 87036 Rende (CS), Italy; ^2^Istituto di Cristallografia, CNR, via G. Amendola 122/o, 70126 Bari, Italy; ^3^Istituto per lo Studio dei Materiali Nanostrutturati, CNR, via U. La Malfa 153, 90146 Palermo, Italy; ^4^Istituto per la Bioeconomia, CNR, via Madonna del Piano 10, 50019 Sesto Fiorentino (FI), Italy*

**Corresponding authors*

E-mail: enrica.fontananova@cnr.it; E-mail: daria.talarico@cnr.it

E-mail: rosaria.ciriminna@cnr.it; E-mail: mario.pagliaro@cnr.it

E-mail: teresa.sibillano@cnr.it; cinzia.giannini@cnr.it

**Supporting information**

**
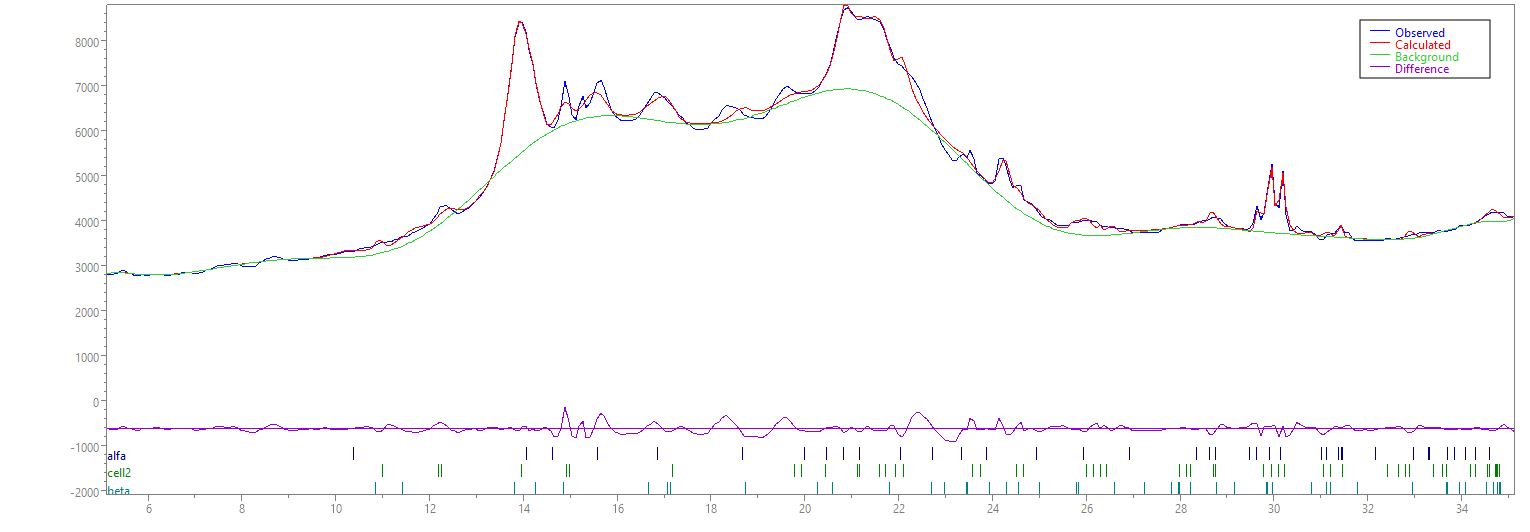
**

**Figure S1**. Calculated and experimental WAXS profiles of lemon CytroCell sourced via hydrodynamic cavitation.

The calculated profile uses selected cellulose crystalline phases.
